# Genome-wide subcellular protein map for the flagellate parasite *Trypanosoma brucei*

**DOI:** 10.1038/s41564-022-01295-6

**Published:** 2023-02-20

**Authors:** Karen Billington, Clare Halliday, Ross Madden, Philip Dyer, Amy Rachel Barker, Flávia Fernandes Moreira-Leite, Mark Carrington, Sue Vaughan, Christiane Hertz-Fowler, Samuel Dean, Jack Daniel Sunter, Richard John Wheeler, Keith Gull

**Affiliations:** 1 Sir William Dunn School of Pathology, University of Oxford, Oxford, UK; 2 Department of Biological and Medical Sciences, Oxford Brookes University, Oxford, UK; 3 Department of Biochemistry, University of Cambridge, Cambridge, UK; 4 Institute of Integrative Biology, University of Liverpool, Liverpool, UK; 5 Division of Biomedical Sciences, Warwick Medical School, University of Warwick, Coventry, UK; 6 Peter Medawar Building for Pathogen Research, Nuffield Department of Medicine, University of Oxford, Oxford, UK; 7 Present Address: Head of Directed Activity Discovery Research, Wellcome Trust, London, UK

**Keywords:** Parasitology, Molecular biology, Organelles, Pathogens, High-throughput screening

## Abstract

*Trypanosoma* *brucei* is a model trypanosomatid, an important group of human, animal and plant unicellular parasites. Understanding their complex cell architecture and life cycle is challenging because, as with most eukaryotic microbes, ~50% of genome-encoded proteins have completely unknown functions. Here, using fluorescence microscopy and cell lines expressing endogenously tagged proteins, we mapped the subcellular localization of 89% of the *T.* *brucei* proteome, a resource we call TrypTag. We provide clues to function and define lineage-specific organelle adaptations for parasitism, mapping the ultraconserved cellular architecture of eukaryotes, including the first comprehensive ‘cartographic’ analysis of the eukaryotic flagellum, which is vital for morphogenesis and pathology. To demonstrate the power of this resource, we identify novel organelle subdomains and changes in molecular composition through the cell cycle. TrypTag is a transformative resource, important for hypothesis generation for both eukaryotic evolutionary molecular cell biology and fundamental parasite cell biology.

## Abstract (web summary)

Eighty-nine percent of the *Trypanosoma* *brucei* proteome is mapped using fluorescence microscopy and cell lines expressing endogenously tagged proteins, and presented in a resource for the community named TrypTag.org.

## Main

Abundant genome data have transformed the molecular cell biology of parasites and model organisms, yet there are still many cryptic genes even in the best studied. Ascribing subcellular localization of proteins assists understanding function and has largely been addressed through ‘omic’ approaches, such as proteomics of purified organelles and hyperplexed organelle localizations by isotope tagging^1^. However, such localization attributions are limited by the accuracy of organelle purification or fractionation, and sensitivity is limited by protein abundance and characteristics. Therefore, microscopy remains the gold standard approach for understanding a protein’s localization and dynamics.

A localization map achieved by high-resolution microscopy enables the study of small, rare or difficult-to-isolate structures, and allows analysis of cell-cycle-dependent changes. This was a transformative resource for studying *Saccharomyces* *cerevisiae*^2^, *Schizosaccharomyces* *pombe*^3^ and human cell lines^4^. Similar protein positional information in a divergent unicellular parasite would provide powerful hypothesis-driven opportunities.

*Trypanosoma* *brucei* is a flagellate unicellular parasite, causing African trypanosomasis in humans and cattle. It is one of a family of important insect-transmitted pathogens, including the human parasites *Leishmania* spp. (leishmaniasis) and *Trypanosoma* *cruzi* (Chagas disease), and a range of animal and plant parasites. The complex *T*. *brucei* life cycle alternates between vector and host with multiple developmental forms and adaptations, including characteristic morphologies and specialized surface antigens^5,6^. The flagellum has multiple functions, including motility, attachment and environmental sensing^7,8^. Moreover, the flagellum is also a widely conserved organelle in eukaryotes and a defining feature of the last eukaryotic common ancestor^9^, but not yet analysed by genome-wide protein localization mapping using microscopy.

**Fig. 1:**
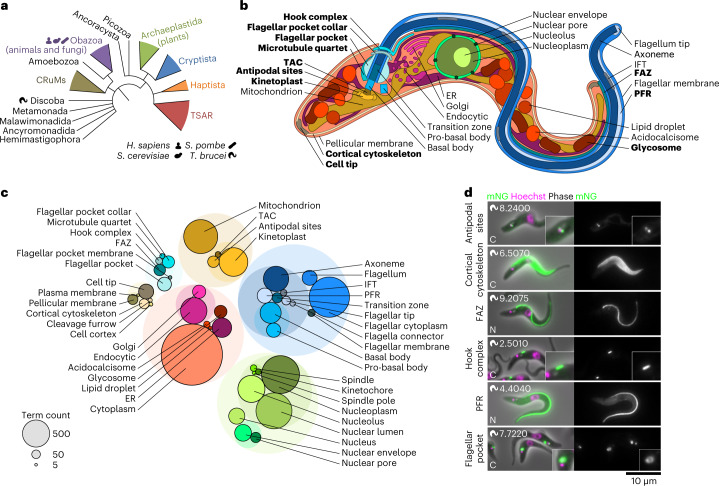
The subcellular protein atlas of *Trypanosoma* *brucei*. **a**, The position of *T.* *brucei* in a simplified phylogeny of eukaryotic life, redrawn from Burki et al.^100^. The human and yeast icons are used throughout to indicate when a protein has an orthologue in these species. TSAR, Telonemia, Stramenopiles, Alveolata and Rhizaria. **b**, The structure of the *T.* *brucei* cell. Each labelled organelle/structure is distinguishable by light microscopy. Further structures associated with cell division are also distinguishable. Organelles unique to, or with notable elaborations in, the *T.* *brucei* lineage are shown in bold. **c**, Number of proteins annotated with each annotation term, giving a representation of the relative complexity of each *T.* *brucei* organelle. Transparent circles represent grouping of annotation terms in an ontology hierarchy. This organelle colour key is used throughout all figures. **d**, Examples of previously uncharacterized proteins localizing to different organelles either unique to or with notable elaborations in the *T.* *brucei* lineage, representative of the quality of microscopy data. *T.* *brucei* TREU927 gene IDs (minus the Tb927. prefix) are shown in the top left and the terminus of endogenous tagging in the bottom left. Source data

*T*. *brucei* is an early-branching eukaryote (Fig. 1a), giving enormous insight into eukaryote evolutionary cell biology and losses or gains in organelle complexity since the last eukaryotic common ancestor. The highly organized cell, with single copies of many organelles (Fig. 1b) and a precise division process^10^, allows unambiguous assignment of cell cycle stages and identification of old and new organelles during and after replication. Protein localization offers insights into organelle subdomains/dynamics and cell-cycle-dependent localization changes.

In this Resource, we generate a high-quality and comprehensive genome-wide protein localization resource in *T.* *brucei*. We demonstrate the power of this resource for understanding microbial evolution and provide insights into the evolutionary cell biology of eukaryotes and their organelles, showing that the gain of kinetoplastid parasitism is associated with increased morphogenetic and cell surface complexity. This powerful single-cell dataset also identifies cell cycle stage-specific and organelle subdomain specializations associated with key parasite functions, including a novel protein important for closed mitosis. The biological knowledge embedded in this dataset is a valuable basis enabling future studies.

## Results

### A high-coverage subcellular localization resource

**Extended Data Fig. 1:**
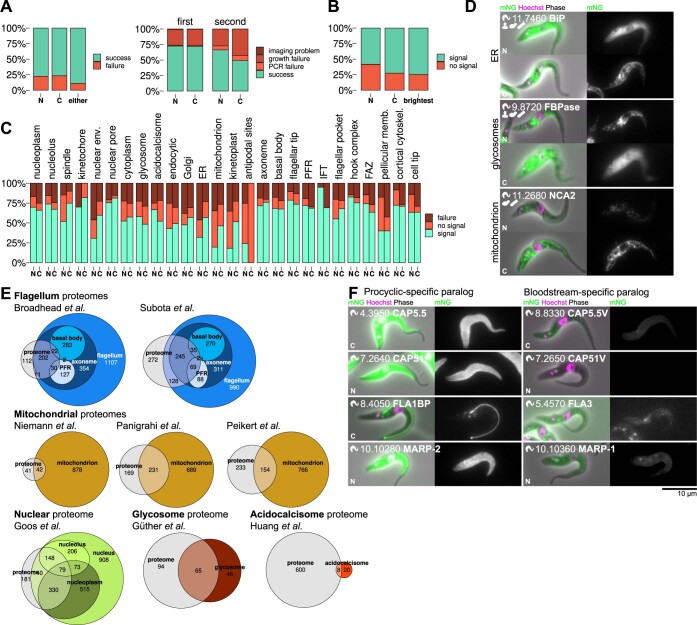
Localisation success rates and reliability. **a**. Left, the proportion of proteins for which a tagged cell line and microscopy data was successfully generated from N-terminal tagging, C-terminal tagging and from at least one (either) terminus. Right, the success rates from the first attempt and a repeat attempt at endogenous tagging, leading to the overall success rate on the left. **b**. The proportion of proteins with detectable signal from N-terminal tagging or C-terminal tagging and from at least one terminus (brightest signal), used to assign a protein localisation. **c**. Consensus from N and C-terminal tagging, broken down by organelle. For each organelle, N indicates the localisation determined by N-terminal tagging of a protein which localised to that organelle by C-terminal tagging, and vice versa for C. **d**. Examples of known proteins with known targeting sequences with a mismatch in localisation or lack of signal when tagged at one terminus: BiP, signal peptide disrupted by N-terminal tagging. FBPase, glycosomal targeting sequence disrupted by C-terminal tagging. NCA2, mitochondrial presequence disrupted by N-terminal tagging. Icons in the top right indicate whether an ortholog is present in humans or yeast (*S. cerevisiae* or *S. pombe*). **e**. Euler diagrams of agreement of mass spectrometry-based high confidence proteomes^17,101–107^. with our protein localisations data, with localisations further broken down by organelle substructures for the flagellum and nucleus. **f**. Examples of known paralogous pairs of proteins with one highly expressed in the procyclic form and one in the bloodstream form^22,108–110^, shown in pairs with the procyclic-specific protein on the left. Both images in each pair are shown with equal contrast. Source data

The *T.* *brucei* genome encodes 8,721 proteins, excluding the variant surface glycoproteins (VSGs, used for antigenic variation) and identical duplicated genes^11^. We generated cell lines and microscopy data in the procyclic life cycle stage by endogenous N- or C-terminal tagging, using the workflow outlined in the project announcement^12^. This resulted in localization annotations for 89% (7,766) of proteins tagged on at least one terminus, and >75% tagged at both (Extended Data Fig. 1a), with ≳250 cells imaged per cell line. Most cell lines had an operationally convincing localization (Methods and Extended Data Fig. 1b): 73% of C-terminal and 59% of N-terminal tagging gave fluorescence signal greater than background intensity and/or in a position dissimilar to background fluorescence (Extended Data Fig. 1b). N- or C-termini refractory to tagging correlated with known targeting sequences; tagging failures are therefore often biologically informative (Extended Data Fig. 1c,d). Manual annotation of protein localization using an ontology (Fig. 1b and Supplementary Tables 1 and 2) yielded a localization database (Supplementary Table 3). Overall, 5,806 (>75% of successfully tagged proteins) had a clear signal (Extended Data Fig. 1a,b), making this a high-coverage transformative resource.

This resource maps the protein composition of all organelles (Fig. 1c). The most complex were the mitochondrion, nucleus and flagellum, although small organelles could also be complex (for example, basal body—307 proteins). Contrastingly, the mitotic spindle was simple (30 proteins). *T*. *brucei* is an early-branching eukaryote (Fig. 1a), where similarities to its host reflect conserved eukaryotic cell biology^13^ and dissimilarities reflect lineage-specific or parasitism-associated adaptations. Structures that are highly adapted to, or found only in, *T*. *brucei* and related parasites (examples in Fig. 1d) could also be complex, particularly the flagellar pocket (the specialized site of endo- and exocytosis^14^) and cytoskeleton. For many proteins in these structures, this is the first indication of potential function and this divergent biology presents potential drug targets.

### Parasite-specific and general eukaryotic features

**Fig. 2:**
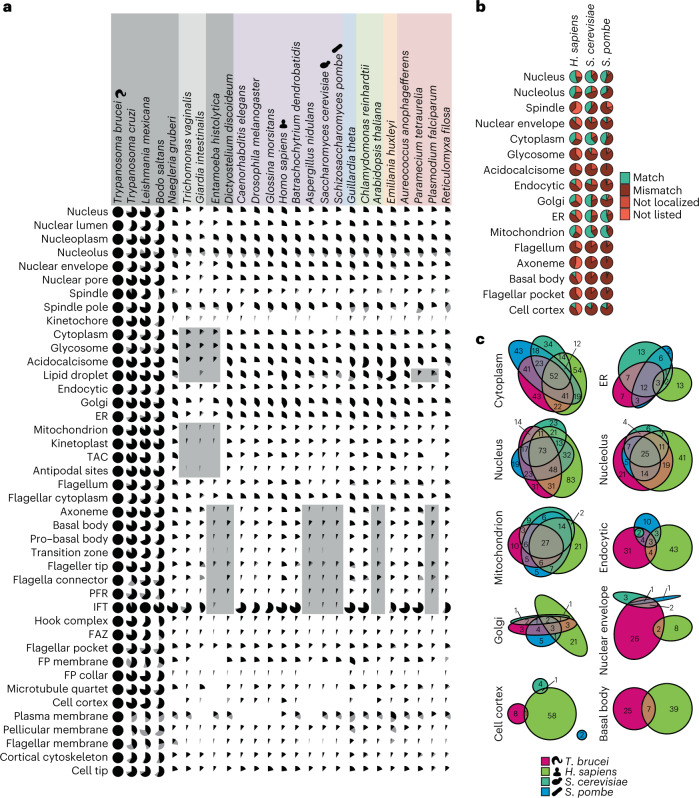
Mapping eukaryote-wide conserved and parasite-specific features. **a**, Presence of orthologues of *T.* *brucei* proteins, grouped by organelle, across eukaryotic life. Pies represent the proportion of proteins with a reciprocal best BLAST (RBB, black) or not an RBB but at least one orthogroup member (grey) in each species. Extended version in Extended Data Fig. 3. **b**, Conservation of localization for proteins with an orthologue in humans or yeast (both budding and fission), broken down by organelle, with human or yeast annotations mapped to our most similar *T*. *brucei* term. Some localizations cannot match (for example, flagellum) as yeast cells lack the structure and cilia were not annotated in human cells. **c**, Euler diagrams showing the degree of agreement of localization of the proteins with a single orthologue in human, yeast and *T*. *brucei* cells or, for the basal body, just humans and *T*. *brucei*. Source data

**Extended Data Fig. 3:**
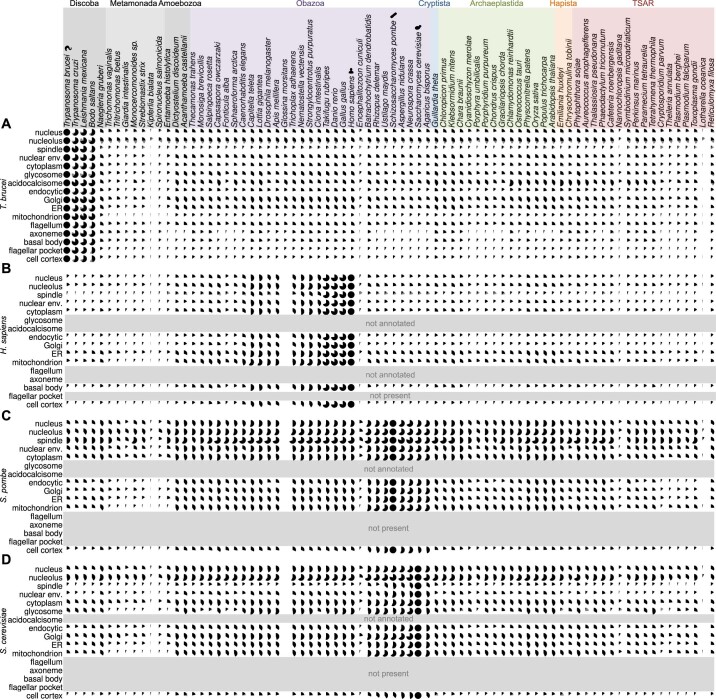
Extended mapping of the universally conserved features of eukaryotic organelles, relative to *T. brucei*, humans and yeast. Complementary version of Fig. 2a. **a**. Presence of orthologs of *T. brucei* proteins, grouped by organelle, across eukaryotic life, using a simplified set of localisation annotations. Pies represent the proportion of proteins with a reciprocal best BLAST (RBB, black) or not an RBB but at least one orthogroup member (grey) in each species. **b-d**. As for A, but plotting orthologs of human proteins (B), *S. pombe* (C) and *S. cerevisiae* (D) proteins, grouped by localisation in their respective genome wide protein localisation projects using a set of terms comparable to A. Source data

**Extended Data Fig. 4:**
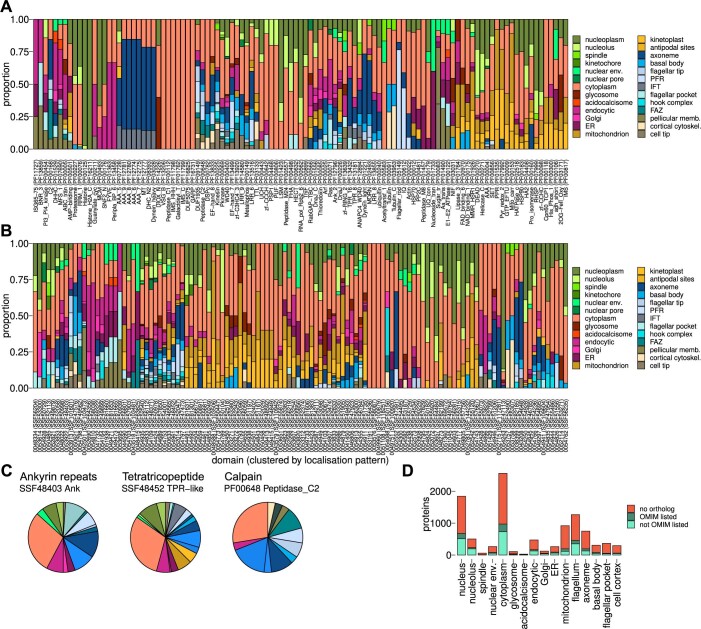
Protein domains and conserved proteins implicated in human genetic disease. **a**. Localisation annotations for all proteins with annotated PFAM domains, grouped by PFAM domain for each domain found in at least 10 proteins. **b**. As for A, except for Superfamily protein domains. **c**. Pie chart representation of Superfamily and PFAM domains for domains highlighted in the main text, using the same colour key as A, B. **d**. *T. brucei* proteins with a human ortholog, summarised by where the *T. brucei* protein localises and whether the human ortholog is listed as involved in human genetic disease in OMIM. Source data

*T*. *brucei* proteins with orthologues across a diverse set of eukaryotes (Supplementary Tables 3 and 4) are the extremely well-conserved core organelle machinery (Fig. 2a and Extended Data Figs. 3 and 4). The nucleus and other membrane-bound organelles (glycosomes or peroxisomes, acidocalcisomes, endoplasmic reticulum (ER) and Golgi apparatus) had a high proportion of conserved proteins (~25%). The flagellum and mitochondrion had a lower proportion—probably reflecting evolutionary innovation in the *T*. *brucei* lineage. This also identified eukaryotes that have lost ancestral features. Some (mostly parasitic, for example, *Plasmodium* *falciparum*) species lacked many orthologues of *T*. *brucei* acidocalcisome or lipid droplet proteins, pointing to reduced or differing ion homeostasis and lipid metabolism.

Orthologues to *T*. *brucei* proteins in different species may act in a different compartment; therefore, localization also indicates if function may also be conserved. Using human and yeast localization data^2–4^, we asked whether human and yeast orthologues of *T*. *brucei* proteins localized to the same organelle (Supplementary Table 5 and Fig. 2b). For proteins with a single orthologue in all four species, we also asked which orthologues had the same localization in all four species (Fig. 2c). The nucleus and mitochondrion have many conserved proteins with conserved localization (~50% localization conservation) (Fig. 2b); however, many organelles do not. As expected, the *T*. *brucei*, human and yeast cell cortex, surface and cell wall differ greatly. Furthermore, human and yeast orthologues of *T*. *brucei* basal body or centriole, spindle, nuclear envelope and endocytic system proteins tend to have differing localizations (Fig. 2b,c). This speaks to the vital core nuclear and mitochondrial biochemistry across eukaryotes, yet evolutionary adaptability of the endomembrane system, probably commensurate with the *T*. *brucei* secretory cargo and the specific cell cortex that is vital for host–parasite interactions.

Change in localization presents an opportunity for adaptation; proteins with conserved domains readily assemble into lineage-specific structures, such as the extra-axonemal paraflagellar rod^15^ (Fig. 2a). Specific protein families tended to have multiple paralogues with diverse localizations, including calpain-like peptidases, tetratricopeptide and ankyrin repeats (Extended Data Fig. 4a–c).

This is the first genome-wide protein localization resource mapping the flagellum/cilium—a complex ancestral eukaryotic organelle necessary for *T*. *brucei* morphogenesis and pathogenicity^8,16^. The conserved axoneme architecture and existing flagellar proteomes^17^ point to high evolutionary conservation; therefore, our high-resolution map of the flagellum will be informative for most flagellates, including pathogens such as *Giardia* and *Trichomonas*. This is also important in humans. Flagellar or ciliary gene mutations are associated with ciliopathies^18^, and of the ~200 axoneme and basal body *T*. *brucei* proteins with a human orthologue, the majority are not yet identified as genetic disease associated (Extended Data Fig. 4d).

### Recent evolutionary innovations associated with parasitism

**Fig. 3:**
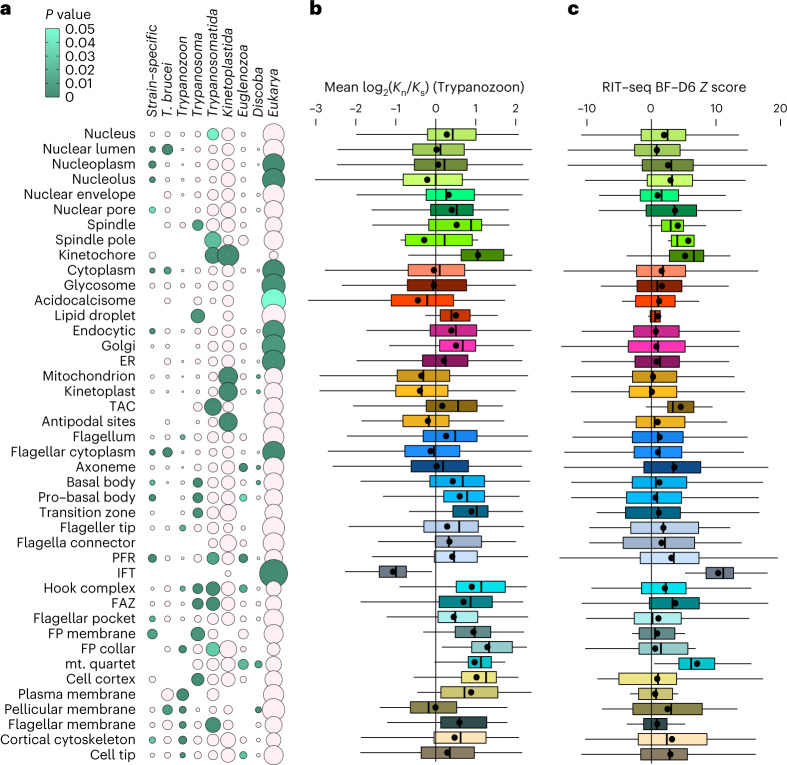
Evolutionary tempos of different organelles reflecting adaptation for parasitism. **a**, Evolutionary distances at which different organelles gained complexity. Circle size represents the proportion of proteins localizing to each organelle that have an orthologue (RBB) in at least one species at that evolutionary distance from *T*. *brucei* 927 and no detectable orthologue in more distantly related species. Green circles indicate a disproportionately large proportion of organelle complexity gained in or retained from the common ancestor of that evolutionary distance, *P* < 0.05 one-tailed hypergeometric test with no adjustment for multiple comparisons. **b**, Ratio of non-synonymous (*K*_n_) to synonymous mutations (*K*_s_) between *T*. *brucei* 927 and other African trypanosomes (Trypanozoon) for proteins with a single orthologue, broken down by organelle. Among these species, amino acid sequence identity is ~50%. **c**. High-throughput fitness phenotype score from ref. ^24^, the result of RNAi knockdown and 6 days in culture as bloodstream forms (BFs), broken down by organelle. Higher values indicate greater fitness cost. Point represents the mean; box and whiskers represent the quartile ranges and the 5th and 95th percentile. *n* = number of proteins annotated with that localization (Supplementary Table 3), analysed as defined in Online Methods. RIT-seq, RNA interference (RNAi) target sequencing. Source data

Determining when protein complexity was gained in each organelle allows the mapping of where and when parasitism-associated adaptations occurred, achieved by identifying the most divergent species with a detectable orthologue of each *T*. *brucei* protein, grouped by localization (Fig. 3a). Most organelle complexity is either shared eukaryote wide or arose around divergence of the class Kinetoplastida. Almost all organelles had large gains in complexity, disproportionately so for the mitochondrion, probably associated with evolution of the kinetoplast (mitochondrial DNA structure). The unusual spindle and kinetochore also emerged at this time^19^. Kinetoplastida includes parasitic and free-living species^20^, so this evolutionary innovation was not exclusively parasitism associated.

Later adaptations coincident with the evolution of parasitism among Kinetoplastida^21^ can now be mapped to organelles. Recent gain in organelle complexity around the evolution of parasitism (Trypanosomatida) and dixenous parasitism (*Trypanosoma*)^20^ (Fig. 3a) correlated with a ratio of >1 for gene non-synonymous and synonymous mutations (indicating selection for new traits), calculated among closely related trypanosomes (*Trypanozoon*) and averaged per organelle (Fig. 3b). The recently adapted organelles are the cell body cytoskeleton, plasma membrane domains and the mitotic spindle—new support for links between cytoskeleton-mediated morphogenesis and parasitism^14,22,23^.

Genome-wide *T*. *brucei* RNA interference (RNAi) mutant fitness^24^, averaged per organelle (Fig. 3c), identified which organelles are most essential with least redundancy. Both evolutionarily ancient and recent structures are essential*:* the mitotic spindle and kinetochores (recent), the tripartite attachment complex (TAC, kinetoplast-associated, recent), intraflagellar transport (IFT, ancient) and the microtubule quartet and flagellum attachment zone (MtQ and FAZ, recent). These structures are linked with the three subcycles that underlie the *T*. *brucei* cell cycle^10^: nuclear DNA replication and segregation (spindle and kinetochore), kinetoplast DNA replication and segregation (TAC), and flagellum-dependent cytoskeletal growth and division (IFT, MtQ and FAZ). Proteins in these structures are therefore high-priority drug targets, exemplified by the recent success of a kinetochore kinase inhibitor^25^. These are on average the most essential organelles; however, parasitism-associated adaptations are probably not limited to them.

### Candidate regulators of cell cycle-dependent morphogenesis

**Fig. 4:**
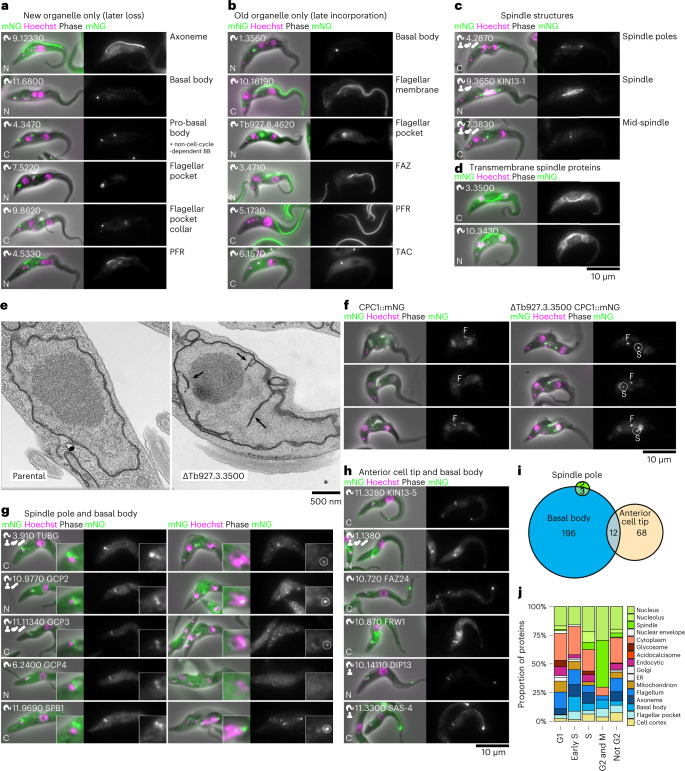
Cell-cycle-dependent organelle composition identifies division and morphogenesis factors. **a**, Six examples of proteins that were found more strongly in the new copy of an organelle, showing cells towards the end of the cell cycle (2K1N and 2K2N). BB, basal body. **b**, Six examples of proteins that were found more strongly in the old copy of organelles, showing cells towards the end of the cell cycle. **c**, Examples of proteins localizing to different spindle structures. **d**, Both proteins localizing to the spindle with predicted transmembrane domains. **e**, Thin-section transmission electron micrographs of the nucleus of parental in comparison with Tb927.3.3500 (CMP1) deletion mutant cells showed abnormal intranuclear membrane never seen in parental cells (arrows) in 42% of nuclei, *n* *=* 38 cells from one clonal cell line. **f**, CPC1::mNG localization in cytokinetic cells in parental in comparison to CMP1 deletion mutant cells shows the normal cleavage furrow (**f**) signal and an abnormal additional cytoplasmic point never seen in parental cells (S, probably the mid-spindle remnant) in 76% of cytokinetic cells, *n* = 17 cells from one clonal cell line. **g**, Localization of the proteins which localize to both the spindle pole (spindle nucleating) and basal body or pro-basal body (axoneme nucleating). GCP4 was not detectable at the spindle pole but was included for completeness of the gamma tubulin ring complex. **h**, Localization of the six proteins that localize to both the basal body or pro-basal body (axoneme nucleating) and the cell anterior tip (hypothetically cortical cytoskeleton nucleating) and not to other structures. **i**, Euler diagram of proteins that localize to the axoneme, spindle and cortical cytoskeleton nucleating structures. **j**, Localization of proteins identified as upregulated at the protein level in particular cell cycle stages by Crozier et al.^43^. Source data

Organelle growth, maturation and size regulation are major cell biology questions^26^, and factors necessary for these processes are probably important for parasite replication and hence potential targets for intervention. The precisely organized *T*. *brucei* cell (Fig. 1b) and cell cycle means each stage is identifiable from morphology^27^. Our resource therefore allows genome-wide molecular-level analysis of organellar growth, duplication and associated protein dynamics. We found known^17,28^ and many novel proteins localizing specifically to either the newly forming or growing (Fig. 4a) or the old or mature (Fig. 4b) copy of almost all cytoskeletal structures. A different proteomic composition when growing versus mature is therefore a fundamental property of cytoskeletal organelles. These proteins may enable templated assembly of daughter organelles, enable or promote organelle growth, prevent growth, stabilize an assembled structure or confer new functions once mature.

The flagellum and associated structures are critical for *T*. *brucei* morphogenesis. Proteins specific to the new flagellum axoneme and the distal new FAZ were particularly numerous. We confirmed known^29,30^ and identified many novel proteins. Interestingly, key cell cycle regulators^29,31^ localize to the distal FAZ. We also identified flagellar or flagellar pocket membrane proteins specific to either the old or the new flagellum (Fig. 4a,b). Protein sorting to plasma membrane domains is therefore sensitive to the maturity of the associated cytoskeleton. This may confer functional differences to cells inheriting the old or new flagellum, which is important as *T*. *brucei* life cycle stage transitions are associated with specialized ‘differentiation divisions’^32^.

Mitosis, cytokinesis and mitochondrial inheritance, effected through attachment of the kinetoplast to the basal body via the TAC, depend on microtubules^10^. The multiple microtubule organizing centres (MTOCs) are presumably important division process regulators. We identified 307 proteins in the basal body—the MTOC for the flagellum—including 12 of the 15 well-conserved core proteins^33^. The basal body complexity perhaps reflects its potential as a master regulator of the cell cycle^34^ and includes ten kinases and three phosphatases, one of which (Tb927.3.690) has previously been identified as important for division^35^.

The unusual *T*. *brucei* chromosomal organization includes 11 megabase chromosomes in addition to many mini and intermediate chromosomes. These additional chromosomes encode part of the critical VSG gene library for antigenic variation; however, little is known about their segregation. The nucleus undergoes closed mitosis, with the spindle MTOC, to which few proteins localized, not associated with the basal body. We identified 14 novel spindle-associated proteins^36^. These included one (Tb927.4.2870) novel spindle pole protein (Fig. 4c), while the remaining few are known (the γ-tubulin ring complex, γTuRC, MLP2 and SPB1^36–38^), constraining possible mechanisms for intermediate or mini-chromosome segregation. Two novel spindle proteins (Tb927.10.3430 and Tb927.3.3500) had transmembrane domains (Fig. 4d).

**Extended Data Fig. 5:**
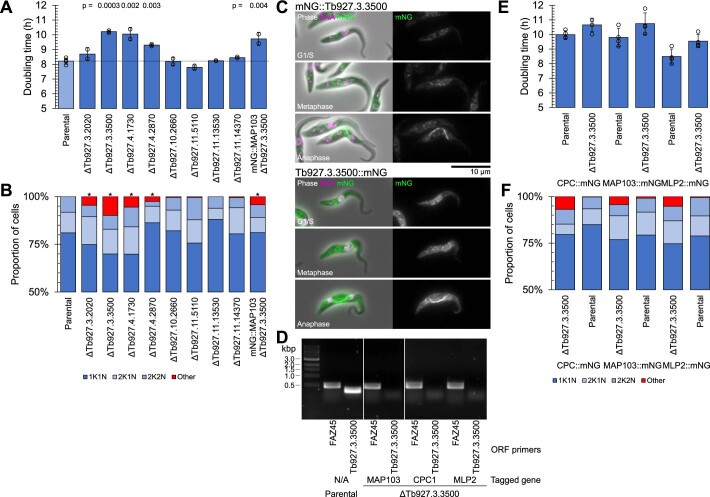
Tb927.3.3500 (CMP1) is a transmembrane spindle-associated protein necessary for normal cell division. **a**. Screening for defects in population doubling time in exponential growth for 8 deletion mutants of novel spindle proteins. Doubling time calculated from *n* = 2 or 3 24 h intervals from one clonal cell line, bars and error bars represent mean and standard deviation, circles represent individual 24 h measurement intervals. *p* values (two tailed T test) relative to parental shown when *p* < 0.05. **b**. Proportion of cells with different numbers of kinetoplasts (K) or nuclei (N) in exponential growth for the cell lines in **A**. Normal cell cycle stages are sequentially 1K1N, 2K1N, 2K2N. For all, ‘Other’ was predominantly 1K0N cytoplasts. *n* = 1 replicate from one clonal cell line. * indicates *p* < 10^−30^ from χ^2^ test. **c**. Localisation of Tb927.3.3500 by N and C-terminal tagging in G1/S, metaphase and anaphase cells. **d**. Confirmation of gene deletion by PCR from genomic DNA using primers specific to the FAZ45 (positive control) or Tb927.3.3500 open reading frame (ORF). The parental (Tb927 1339) and three independent deletion mutants generated on the background of different tagged spindle proteins are shown (cell lines for **E,F**). **e**. Population doubling time in exponential growth for three independently generated deletion mutants of Tb927.3.3500, on the genetic background of different tagged spindle proteins (CPC1, MAP103, MLP2), calculated from *n* = 4 24 h intervals. *p* = 0.016 from paired two-tailed T test of all deletion mutant relative to their respective parental lines. Bars and error bars represent mean and standard deviation, circles represent individual 24 h measurement intervals. **f**. Proportion of cells with different numbers of kinetoplasts (K) or nuclei (N) for the cell lines in E. *p* < 10^−30^ from χ^2^ of pooled deletion mutants relative to pooled parental lines. Source data

To test the ability of this resource to identify functionally important proteins, we generated deletion mutants of eight novel spindle proteins (Extended Data Fig. 5a,b). *T*. *brucei* procyclic forms lack a checkpoint to inhibit cytokinesis upon mitosis failure, knockout phenotypes including reduced growth rate and anuclear cytoplast (zoid) production are therefore indicative of a potential mitotic function^10^. This knockout phenotype and a late mid-spindle localization (Fig. 4d and Extended Data Fig. 5c) identified Tb927.3.3500 (a protein conserved in trypanosomatids) as important in closed mitosis; thus, we named this protein closed mitosis protein 1 (CMP1). Its transmembrane domains indicate localization to the nuclear envelope around the late spindle. Therefore, we investigated nuclear ultrastructure in a CMP1 deletion mutant using transmission electron microscopy, which revealed extensive abnormal intranuclear membranes (Fig. 4e,f). To ascertain if mitosis defects cause this phenotype, we deleted CMP1 in cell lines expressing tagged spindle proteins: MAP103 (microtubules), MLP2 (poles) and chromosomal passenger complex (CPC1) (Extended Data Fig. 5d). These lines confirmed the growth defect and zoid formation phenotypes (Extended Data Fig. 5e,f). There was no large change to MAP103 or MLP2 localization; however, CPC1 was affected. While CPC1 normally localizes to the middle of the spindle and then the cleavage furrow^39^, in the CMP1 deletion mutant the spindle signal was abnormally persistent and retained during cytokinesis furrow ingression (Fig. 4f). CMP1 is therefore probably necessary for normal resolution of nuclear envelope division and the late spindle, at the end of closed mitosis.

**Extended Data Fig. 6:**
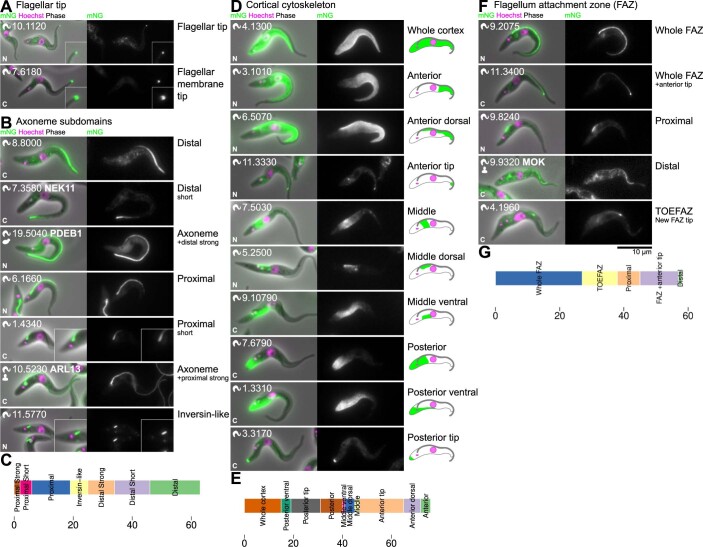
Sub-domains of the flagellar and cellular cytoskeleton implicated in morphogenesis. **a**. Examples of the two major flagellar tip localisations – a small point (axoneme associated) or a horseshoe shape (membrane associated). **b**. Examples of proteins localising to the 7 readily identifiable proximal and distal flagellum, likely axoneme, subdomains. **c**. Number of proteins localising to different proximal and distal domains. **d**. Examples of proteins localising to a range of sub-domains of the microtubule-based cortical cytoskeleton. At least 10 sub-domains, 7 novel, exist. **e**. Number of proteins localising to each cortical cytoskeleton sub-domain. **f**. Examples of proteins localising to a range of sub-domains of the flagellum attachment zone (FAZ) which is a specialised seam in the cortical cytoskeleton required for lateral attachment of the flagellum. At least 5 sub-domains exist. The anterior cell tip and distal FAZ tip appear synonymous. Some FAZ proteins localise only to the tip of the extending FAZ (TOEFAZ). **g**. Number of proteins localising to each FAZ sub-domain. Source data

Trypanosomatids have distinctive morphologies, probably a selective advantage for host and vector interaction, defined by the subpellicular microtubules, a parallel one-layer-thick microtubule corset under the plasma membrane^40^. Microtubule minus ends are found throughout the array; however, where they are nucleated remains unknown: perhaps via dispersed nucleation within the array or via a major anterior MTOC, with subsequent sliding into the array. As previously described^37^, we did not detect γTuRC proteins in the array (Fig. 4g). Interestingly, at the sensitivity we achieved, we identified no proteins shared among all MTOCs. Several proteins, including SAS-4 (Tb927.11.3300), localized to the basal body and the subpellicular array (Fig. 4f), but none also localized to the spindle poles (Fig. 4g–i). We identified many subpellicular array-associated proteins that may contribute to nucleation or organization. Approximately 60 proteins localized to most of the array and many more to one end of the array, at the cell tips (Extended Data Fig. 6). As the tip of the new growing FAZ becomes the site of cytokinetic furrow ingression^41^, this new subpellicular array anterior tip is probably a key MTOC.

Like other eukaryotes, the *T*. *brucei* cell cycle is regulated by cyclins (CYC) and cyclin-related (CRK), aurora (AUK) and mitogen-activated (MAPK) kinases^10^. However, it is incompletely known how these proteins control division of the *T*. *brucei* cell architecture; previous meta-analyses, such as of the spindle assembly checkpoint^42^, were limited to orthologue presence or absence. Our localizations implicate proteins in regulation of division of specific organelles: MAPK4 (Tb927.6.1780) localized to the basal body, MAD2 (Tb927.3.1750) to the microtubule quartet, MAPK6 (Tb927.10.5140) to the cortical cytoskeleton and posterior cell tip, and CRK1 (Tb927.10.7070) to the mitochondrion. CRK1 location maybe particularly important as how the division of the mitochondrion and kinetoplast are coordinated is unknown. Proteins with cell-cycle-dependent abundance^43^ tended to localize to organelles dividing at the corresponding cell cycle stage (Fig. 4j). Few proteins had localizations that changed through the cell cycle, and we only re-identified known examples: AUK1 (Tb927.11.8220), CYC6 (Tb927.11.16720) and AUK3 (Tb927.9.1670) (refs. ^44,45^). These remain the most interesting candidates for master cell cycle regulators.

### Novel organelle subdomains

Specialized functions often occur in specialized organelle subdomains and we discovered subdomains in most *T*. *brucei* organelles. Their presence points to assembly or maintenance processes, as a uniform protein distribution typically reflects random free diffusion.

*T*. *brucei* flagellar-driven motility is critical for virulence in mammalian hosts and development in the fly vector^46,47^. Many cytoskeletal structures, including flagella, have functions associated with specific subdomains^17,29,30,48–50^, and we identified numerous proteins in the axoneme, cortical cytoskeleton and FAZ subdomains (Extended Data Fig. 6). We showed that the flagellum tip was particularly complex (Extended Data Fig. 6a), with 60 proteins localized to either the axoneme or flagellar membrane tip. These are of interest for environmental sensing as *T*. *brucei* swims with the flagellum leading. We re-identified known signalling-associated proteins^51,52^ and discovered several potential signalling factors: casein kinase (CK1, Tb927.3.1630), a META domain-containing protein (Tb927.5.2230), whose *Leishmania* orthologue is a virulence factor^53^, and a sodium/hydrogen antiporter (Tb927.11.840).

*T*. *brucei* life cycle developmental forms have characteristic morphologies, requiring substantial cortical cytoskeleton remodelling. We identified ten cortical cytoskeleton subdomains (Extended Data Fig. 6d,e), greatly extending the previous discovery of a posterior–ventral domain (containing PAVE1, Tb927.8.2030) (ref. ^50^), with particularly complex anterior and posterior tip subdomains. Like the flagellum tip, the cortical cytoskeleton posterior tip maintained its protein cohort over the cell cycle. The posterior tip included known microtubule end-binding or tip-tracking proteins EB1 (Tb927.9.2760) (ref. ^54^) and XMAP215 (Tb927.6.3090) (ref. ^55^) and many kinesins; their dynamics probably maintain its structure. Kinesins, known vital kinetoplastid-specific TOEFAZ/CIF components and proteins also specific to the distal FAZ^29,31^ localized to the anterior tip (Extended Data Fig. 6e). This indicates that the cortical cytoskeleton is more complex and dynamic than previously appreciated.

**Fig. 5:**
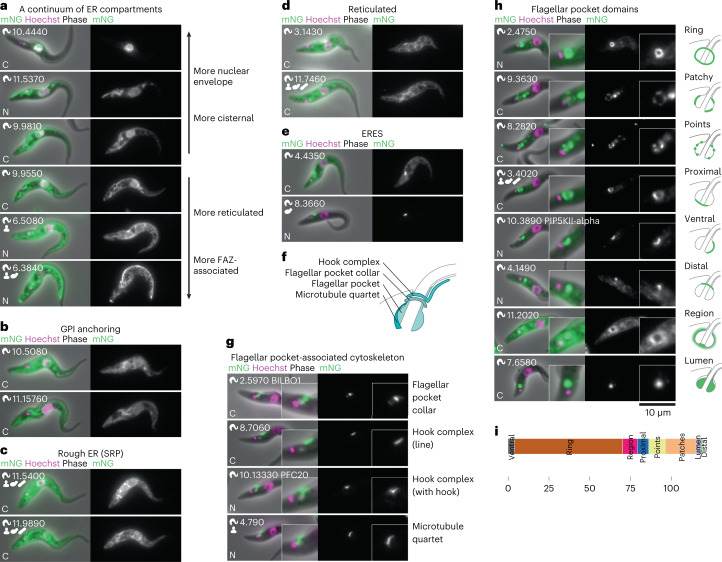
Specialized subdomains of the endomembrane and endo- and exocytic systems. **a**, Examples of proteins localizing to different regions of the ER, which broadly exist as a continuum from more cisternal to more reticulated. **b**, Enzymes for GPI anchoring tend to have a moderately cisternal localization. **c**. Signal recognition particle (SRP) proteins tend to have a moderately reticulated localization corresponding to rough ER. **d**, Examples of proteins with a particularly reticulated localization. **e**, A large subset of proteins also localize strongly to the ERES in addition to the ER. **f**, Cartoon of the cytoskeleton structures associated with flagellar pocket. **g**, Examples of proteins localizing to cytoskeleton structures associated with the flagellar pocket. **h**, Examples of proteins localizing to different flagellar pocket domains and flagellar pocket-associated domains. **i**, Number of proteins localizing to each flagellar pocket subcompartment. Source data

All *T*. *brucei* life cycle stages are extracellular and their host-exposed surface membrane is predominantly covered in glycosylphosphatidylinisotol (GPI)-anchored proteins. Beyond the well-known ER exit site (ERES) and nuclear envelope, further ER subdomains were detected (Fig. 5a–e), probably representing functional specializations. The GPI transamidase complex, necessary for GPI anchoring, and a subset of chaperone proteins, DnaJ46 (Tb927.3.1430) and BiP (Tb927.11.7460), localized to a cisternae-like subdomain (Fig. 5b*,*c). This ER complexity highlights the challenge of understanding how enzyme position is maintained in subdomains as they process a high flux of substrate.

High flux is also necessary for cell surface maintenance, demanding rapid trafficking of material on and off the cell membrane. Complex endo- and exocytic-associated structures are common^56^, and we observed complexity in the associated membrane domains. We identified eight subdomains of the flagellar pocket, which is supported by a complex cytoskeleton (Fig. 5f–i). These pocket subdomains potentially support the high endo- and exocytic flux through this small membrane domain^57^. Many related species also have a cytostome, which probably contributes yet more spatial complexity^41^. We identified two apparently lumenal flagellar pocket proteins (Tb927.7.6580 and Tb927.7.6590), showing that *T*. *brucei* maintains a small, localized extracellular environment.

**Extended Data Fig. 7:**
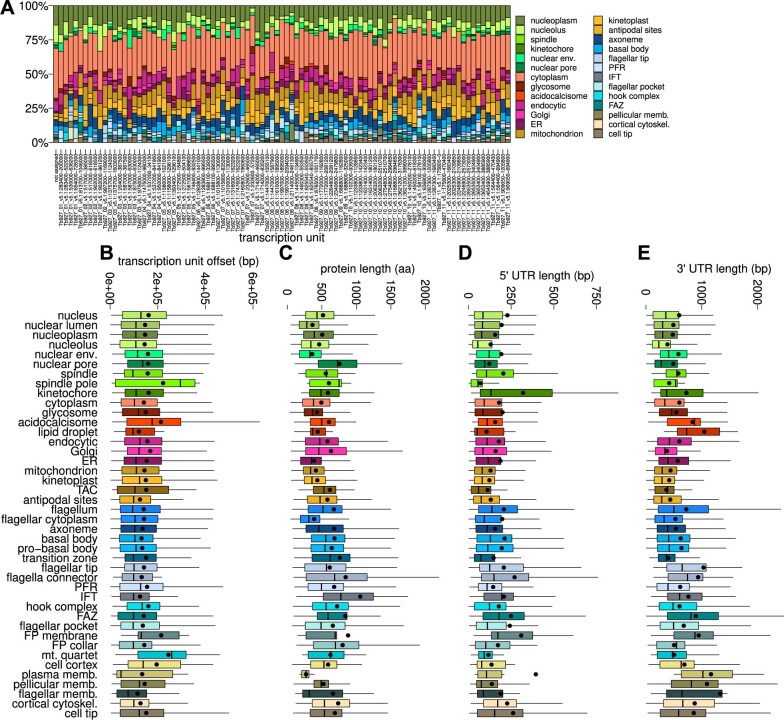
Gene transcription units do not reflect grouping in protein localisation. **a**) Localisation terms for proteins encoded by each transcription unit. **b-e)** Gene properties, broken down by protein localisation. Box represents the median and interquartile range, whiskers represent the 5^th^ and 95^th^ percentile and the dot represents the mean. *n* = number of proteins annotated with that localisation (Supplementary Table 3), analysed as defined in Online methods. B) Offset in base pairs of the start of the gene from the start of the transcription unit. C) Predicted protein size in amino acids. D) 5′ UTR length. E) 3′ UTR length. Source data

**Extended Data Fig. 8:**
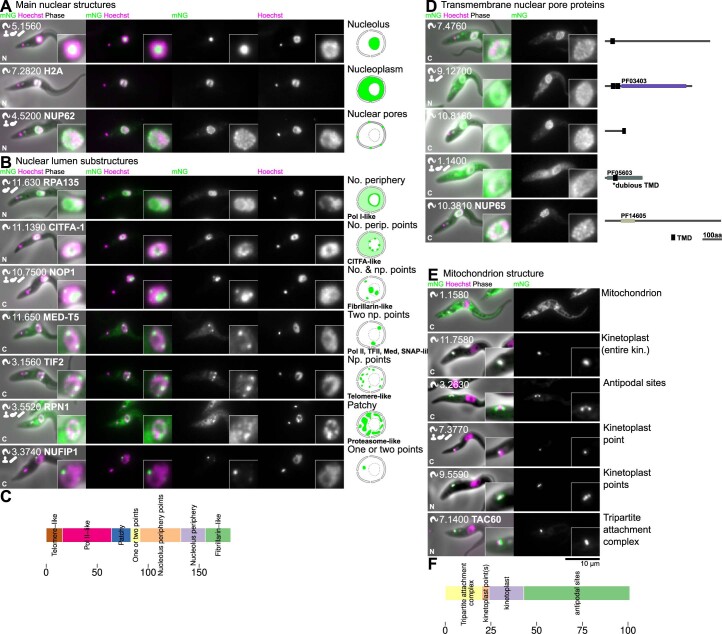
Non-membrane bound complexity of the nuclear and mitochondrial DNA compartments. **a**. Examples of proteins localising to major nuclear subcompartments: the nucleolus, nucleoplasm and the nuclear pores. **b**. Examples of characterised proteins localising to a range of sub-domains within the nuclear lumen. 7 characteristic localisation patterns are visible. Likely functions in Pol I, Pol II, nucleolar (fibrillarin), telomere and nuclear proteasome function are ascribable to other proteins with a similar localisation. No. = nucleolus, np. = nucleoplasm. **c**. Number of proteins localising to each nuclear lumen sub-compartment (excluding simple nucleolar and nucleoplasmic localisations) Pol II-like classification was based on a qualitative similarity to known Pol II proteins. **d**. All five proteins localising to the nuclear pores with predicted transmembrane domains, shown next to a cartoon representation of the protein domain structure. All except NUP65 (Tb927.10.3810) are novel. **e**. Examples of proteins localising to different mitochondrion and kinetoplast structures **f**. Number of proteins localising to each kinetoplast-associated structure. Source data

Gene expression control in *T*. *brucei* is atypical, as co-transcribed gene arrays are processed into mature messenger RNAs by trans-splicing of a 5′ spliced leader and RNA polymerase I (Pol I) is used to transcribe surface coat protein genes. Genes encoding proteins found in particular organelles were neither enriched in specific gene arrays nor at a particular position relative to the transcription start (Extended Data Fig. 7). We identified protein cohorts with characteristic nuclear localization patterns: one, two or multiple points within the nucleoplasm, or nucleolus-associated points (Extended Data Fig. 8a–c). On the basis of previously characterized proteins, these patterns are probably associated with Pol II factories for spliced leader transcription^58^, telomeres^59^, fibrillarin-like (potential Cajal bodies)^60^ and NUFIP bodies^61^. Proteins with a similar localization will probably have associated functions. Similarly, the nucleolus periphery was enriched in *T*. *brucei* Pol I subunits (RPA proteins, uniform signal) and basal Pol I transcription factors (CIFTA proteins, punctate signal). Overall, the organization of the trypanosome nucleolus differs from that of metazoa^62^.

We found the mitochondrion was uniform in composition. However, the kinetoplast region appeared complex (Extended Data Fig. 8e,f), with known and novel proteins that localized to the antipodal sites (associated with DNA replication)^63^, localized to the TAC^64,65^ and co-localized with kinetoplast DNA^66^. We also identified novel kinetoplast-associated foci, although their function is unknown.

### Cell posterior is a complex site of protein moonlighting

**Fig. 6:**
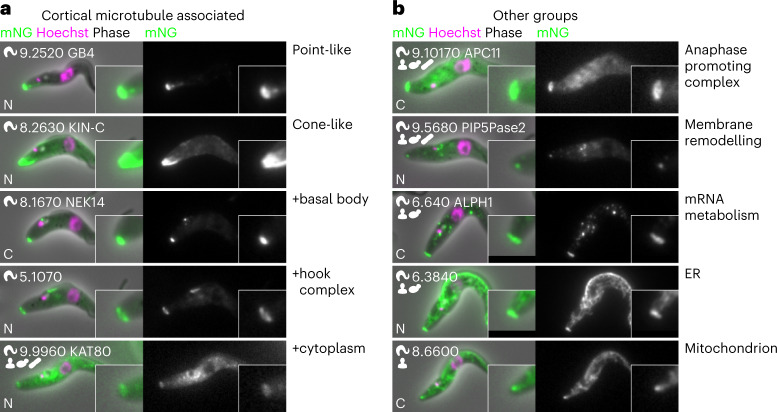
Proteins involved in diverse processes also localize to the posterior cell tip. **a**, Many proteins that localize to the posterior tip are plausibly microtubule associated and a subset also localize to other microtubule-containing structures. **b**, Proteins with diverse predicted functions strongly localize to the cell posterior tip, with some also localizing to other structures in the cell.

Our genome-wide *T*. *brucei* protein localizations revealed an eukaryote with many normal organelles, but distinctive specializations. However, the posterior cell tip stood out as a structure of unexpectedly high complexity (Fig. 6). Many proteins localized specifically there, while also being a common second site for proteins localizing to another organelle or structure. This may be a ‘moonlighting’ localization in addition to the expected or known site of protein function.

The posterior tip therefore has links with several organelles. First, many microtubule-associated proteins localized to various cytoskeletal structures and the posterior tip (Fig. 6a). Second, endo- or exocytic proteins (including clathrin), often localized to the flagellar pocket and a focus at the posterior tip, perhaps an alternative site of import or export or responsible for membrane remodelling. Third, ER or mitochondrion proteins (including DLP1) with an additional focus at the posterior tip, a potential ER or mitochondrion–posterior interaction or membrane management site (Fig. 6b). Fourth, proteins probably involved in RNA catabolism, including two known (XRNA^67^ and ALPH1 (ref. ^68^)) and seven novel, which localized to RNA granules with an additional posterior tip focus. Finally, nine out of ten anaphase promoting complex (APC) proteins^69^ and the APC-interacting kinetochore protein KKT10 (Tb927.11.12410)^70^ localized to the posterior cell tip and the mitotic nucleus (Fig. 6b). The functional importance of this is unclear: is the cell posterior just a common site for sequestration? However, the proteins present suggest cell cycle-dependent membrane management for abscission of the plasma, ER and mitochondrial membranes.

## Discussion

Protein localization—and the timing of localization—is central to cell function, defining the site of action of a protein, the substrates or interaction partners available and when they may interact. Determining localization from microscopy is powerful, being high-content single-cell data. Our mapping of the *T*. *brucei* cell comprises not only 7,766 protein localizations but also ~5,000,000 individual cell images, capturing cell cycle and organelle dynamics, available for ongoing analyses.

*T*. *brucei* is a highly structured single-cell organism, illustrative of the enormous diversity among unicellular eukaryotes. Our localization data indicate extensive placement of specific biochemistry at defined sites or organelle subdomains, for example, the complexity of the flagellar pocket membrane or the multiple specializations of the cell posterior. Nonetheless, even in this early-branching lineage, much eukaryotic biology is conserved, thus informing eukaryotic evolution. As improvements in protein structure predictions increase our ability to detect extremely divergent orthologues, *T*. *brucei* will become even more informative for understanding fundamental biology and pathogenic specializations.

Some specializations reflect adaptations for parasitism, and represent streamlining or examples of ‘extreme biology’ where normal structures become highly elaborated. We show recent morphology-associated adaptations in the cytoskeleton, especially those associated with the flagellar pocket, defining the molecular machinery underlying the characteristic trypanosomatid parasite morphologies. Our data hint at new adaptation themes. Even in the procyclic form, not directly exposed to an adaptive immune system, the exposed cell surface is simple compared with the flagellar pocket—perhaps some environmental sensation occurs in the flagellar pocket and endocytic system? The spindle has undergone many recent changes—and we show a novel trypanosomatid-specific protein is necessary for closed mitosis. This opens up a new area of research on nuclear envelope resolution in a closed mitotic system. Is further spindle adaptation associated with the evolution of the VSG-containing minichromosomes? Our localization database is a key resource for further work to understand adaptation for parasitism.

Protein localization has an intrinsic limitation that the protein must be expressed to be localized. Our data report the procyclic form expression programme. This caveat applies to life-cycle-specific expression in other unicellular organisms, such as spores and meiotic stages in yeast or the multitude of life cycle stages in apicomplexa. This is therefore a generic issue for protein localization databases in all systems. For *T*. *brucei*, procyclic non-expression may be evidence that the protein has stage-specific expression. Use of this information identified the first bloodstream form-specific transcription activator for monoallelic antigen expression, ESB1 (ref. ^71^).

Localization may also be influenced by the position of the tag, disrupting cryptic post-translational modifications, targeting sequences or only localize part of the protein if proteolytically cleaved. Also, spatial positions are informative: for example, the large TAC protein p197, where the visibly different but adjacent localizations by N (TAC) and C (basal body and pro-basal body) terminal tagging probably reflect its size and orientation.

Genome-wide protein localization is important for all organelles, particularly in a highly structured cell such as *T*. *brucei*. Our database is important for interpreting how cellular complexity is encoded by a genome and is modulated in space and time. *T*. *brucei* is now the fourth eukaryote, the first eukaryotic pathogen and the first flagellate for which a genome-wide protein localization resource has been constructed. The http://tryptag.org database contains information on all organelles and is an important searchable resource, and the images and associated annotations have been integrated onto VEUPathDB^11^. The addition of an early-branching eukaryote to the other three organisms is of particular value, enhancing our view of general eukaryotic and parasite evolution.

## Methods

### Cell lines, culture and genetic modification

*Trypanosoma* *brucei* *brucei* TREU927 was selected for our protein localization database as it is the original genome strain^72^ with a high-quality reference genome supported by community annotation^11,73^. We used the SmOxP9 line, which expresses T7 polymerase and Tet repressor^74^. The procyclic form (PCF) life cycle stage was used as it is readily grown in culture, grows to high densities and is more amenable to high-throughput tranfection efficiencies. PCFs were grown in SDM79 (ref. ^75^).

Tagging constructs were generated by long primer polymerase chain reaction (PCR) using a standard plasmid (pPOTv4.2) encoding a drug selectable marker (BSR) and a fluorescent protein with GS linkers (mNG), as the template^76^. The 5′ end of the primers contain 80 bases of homology either to the target gene or its 5′ or 3′ untranslated region (UTR) allowing homologous recombination into the target locus, when introduced by electroporation^76^. Following electroporation in 96-well plates, the transfectants were then transferred to four 24-well plates for drug selection of stable transfectants in 2 ml culture medium with 20 µg ml^−1^ blasticidin^12,77^. Once transfectants had grown to ~4 × 10^7^ cells ml^−1^ they were subcultured once in 24 plates to approximately 1 × 10^6^ cells ml^−1^, followed by 24 h growth was used to give a healthy population for microscopy

During high-throughput tagging, success rates (percent success in generating a construct by PCR, selecting a drug resistant cell line and observing a convincing subcellular localization) were monitored (Extended Data Fig. 1a–c), as was agreement with known localizations and existing proteomic data (Extended Data Fig. 1e). Failures were repeated at least once, and as repeats had a comparable success rate to first attempts, appeared largely stochastic (Extended Data Fig. 1a). Some genes were truly refractory, including those with known problematic gene models, for example, Tb927.11.1090 C-terminal tagging consistently failed. This gene model is actually the N terminus of ClpGM6, an extremely large and repetitive gene, with C-terminal ClpGM6 found in the downstream gene model Tb927.11.1100 (ref. ^78^). Such gene model issues were not corrected here.

Microscopy was carried out on live cells. As they are motile, the cells were washed three times with phosphate-buffered saline, which allows them to adhere to glass. Hoechst 33342 (500 ng ml^−1^ was included in the first wash to stain the DNA^79^. Micrographs were captured on a Leica DM5500 B epifluorescence microscope with a 100 W mercury arc lamp and either a 100× or 63× NA 1.4 oil immersion objective (the vast majority at 63×) and an Andor Neo 5.5 sCMOS camera running in 16-bit high well capacity mode, using Micromanager (no single version, updated over the course of data collection)^80^. mNG fluorescence was captured using the L5 filter cube, excitation 480/40 nm, dichroic 505 nm and emission 527/30 nm. A standard exposure time of 2,000 ms was used, unless the mNG signal was particularly bright. Typically, four to five fields of view (aiming for 200 or more cells) were captured, with more when a cell line was identified as having a rare (that is, cell cycle/dependent) signal.

Before analysis, all images were subjected to the same corrections: first, camera amplifier offset per pixel column in the upper and lower halves of the images (measured from no illumination images) were subtracted. Second, the median of all images captured on that day (typically >200) was taken as background signal and used for flat field correction. Then, finally, scaling of 100× images to 63× equivalent, and normalization of the green channel signal intensity from exposure time and any magnification scaling (2.51×).

Microscope images were manually checked for quality (healthy cell appearance, cell number, appropriate exposure time, focus and so on) and the tagging flagged for repeating if there were substantial quality concerns.

### Gene selection for tagging

Tagging was based on version 5.1 of the genome sequence^72^. The initial target set based on *T*. *brucei* TREU927 genome annotations from TriTrypDB release 5.0 (June 2013). This comprised all genes that mapped to one of the 11 megabase chromosomes (Tb927_01_v5.1 to Tb927_11_v5.1), excluding the chromosome 11 right hand fork (Tb927_11_RH_fork_v5.1) and excluding genes annotated as a VSG or VSG expression site associated genes. The latter are only expressed in the bloodstream form, where VSGs are used for antigenic variation.

New gene models added by TriTrypDB to the megabase chromosomes over the course of the project up to TriTrypDB release 45 (August 2019) were also tagged, as were previously identified transcribed small open reading frames^81^ (which could be unambiguously mapped to the genome) and a small number of manually selected genes not mapped to megabase chromosomes. We attempted tagging of 479 genes whose gene models were removed as unlikely as of the TriTrypDB release 45. Strain-specific proteins tended to localize to the nuclear lumen, cytoplasm and flagellar cytoplasm; this localization can arise spuriously (Extended Data Fig. 1d), and we suspect that these are dominated by incorrect gene models.

Integration of the transfected construct occurs using the endogenous homologous recombination machinery, specificity is therefore conferred by the uniqueness of the homology arms. Generally, only one N- and one C-terminal tagging attempt was made for a set of genes that could not be uniquely targeted. This mostly affected genes that are found tandemly duplicated or in an array.

All genes were tagged at the C terminus, irrespective of whether they had a predicted targeting sequence (for example, glycosomes have a known C-terminal targeting tripeptide^82^). Genes not predicted to have an N-terminal signal peptide (SignalP *P* < 0.5, as indicated by TriTrypDB at the time of primer design) were tagged at the N terminus. Note that this is not a sensitive predictor in *T*. *brucei*.

### Annotation of protein localization

Each cell line was manually annotated by a group of at least three experts using an ontology of 45 annotation terms for different organelles or cell structures, on the basis of consensus of the ≳250 cells imaged per cell line. This extended our previous description of the characteristic appearance of over 30 different organelles and structures^83^. This hierarchical ontology has specific (for example, ‘nucleoplasm’ or ‘axoneme’) and more general terms localizations (for example, ‘nucleus’ or ‘flagellum’). The more general term was used if a localization was ambiguous. If localizing to multiple organelles then all relevant terms were used (that is, additive annotation) (Supplementary Table 1).

We used a further ontology to describe any additional structure within each organelle (for example, ‘patchy’, ‘weak’ or ‘points’)^83^. In some cases, these were used for lower-confidence annotations (for example, nucleus (points) rather than nuclear pores). The ‘weak’ modifier was reserved for localizations with signal comparable to background auto-fluorescence (Supplementary Table 2).

Manual annotation of weak signals were supplemented by automated mNG fluorescence signal intensity, measured from all cells from all images of all cell lines. For reference^4^, independent samples of the parental cell line were grown and prepared for microscopy identically to tagged cell lines. Individual cells were identified, oriented and cropped from the images automatically using intensity thresholding of the phase contrast image after a series of unsharp and background subtraction filters to generate cell masks, as previously described using ImageJ v1.52a (refs. ^84,85^) and mean mNG signal intensity (sensitive to overall signal) and 99th percentile mNG signal intensity (sensitive to small bright structures) calculated. Auto-fluorescence tended to occur in the mitochondrion, cytoplasm and/or endocytic system. Therefore, any mitochondrion, cytoplasm or endocytic system annotation where both mean and 99th percentile green signal intensity were below the parental cell line were automatically given the ‘weak’ modifier. mNG images are displayed mapping black to the median signal outside of cells and mapping white to 4,500 or the maximum pixel value, whichever is higher.

Cell lines were non-clonal, as necessitated by the high throughput. From previously determined transfection efficiency^76^, we estimate that populations were typically derived from 5–20 clones. In some cell lines this leads to heterogeneity, and in these cases organelle annotations were given a modifier of the approximate proportion of the population with the signal.

For all downstream analyses, a protein was listed as a component of an organelle if it was annotated as localizing to that organelle or cell structure, any substructure of that organelle, not annotated ‘weak’ and occurring in at least ~10% of the population. For some analyses, a simplified set of localizations are used. In these cases, proteins were listed as localizing to the nearest parent term in the simplified list.

This database of microscopy data and human annotations can be viewed and downloaded at http://tryptag.org with the annotations and an example image viewable and searchable at http://tritrypdb.org.

### Evaluation of localization reliability

Localizations for a protein from N- and C-terminal tagging represent independent biological replicates, and we typically tagged the N and C terminus genome wide only once. In most (>70%) cases, both termini gave a similar localization (Extended Data Fig. 1c). In the remainder, one terminus tended to give no detectable signal while the other gave a clear localization, as noted in the results this often correlated with a known targeting sequence. Only in a small minority (2%) was there a clear discrepancy (Extended Data Fig. 1c). Therefore, no localizations were excluded from genome-wide analysis.

mRNA UTRs, particularly 3′ UTRs, are implicated in life cycle stage regulation. We tested the impact of 5′ UTR (by N-terminal tagging) and 3′ UTR replacement (by C-terminal tagging) using known life cycle stage-specific paralogous protein pairs, which showed expression levels that correlated with the expected stage specificity despite UTR replacement (Extended Data Fig. 1f). Some cell lines with undetectable signal may represent specific expression of that protein in a different life cycle stage.

We treated two localization types with lower confidence, as they tended to occur as a ‘contaminant’ at low frequency in cell lines: no detectable signal (‘faint’ cells, similar to the parental cell line) or cells with a uniform cytoplasm, nuclear lumen and flagellar cytoplasm signal (‘bright’ cells, similar to cells expressing mNG not fused to a protein). To determine their origin, we cloned and sequenced the modified locus in one ‘faint’ and one ‘bright’ contaminant from otherwise successful tagging. Both had a frame shift originating from the plasmid-binding region of the primer, in both cases introducing an early stop codon. They are therefore stochastic errors probably arising from primer synthesis errors.

### Statistics and reproducibility

All microscopy images representing a cell line show representative cells from images of typically ≳250 cells from one non-clonal population, and all downstream analysis is derived from the annotation of these localizations. This is data from a single replicate, we carried out genome-wide N- and C-terminal tagging only once, and evaluated protein localization reliability as described above.

### *Trypanosomabrucei* protein properties

Gene metadata for analysis were derived from TriTrypDB release 47 (April 2020). This includes gene names and descriptions; genome coordinates; predicted gene mRNA, coding and protein sequences; and basic predicted protein properties (molecular weight, isoelectric point and so on). Predicted functions were derived from predicted PFAM^86^ and SUPERFAMILY^87^ protein domains via TriTrypDB.

Pol II protein-coding gene transcription units were mapped manually. Genomic regions over 50 kbp with non-VSGs genes in a consistent orientation were mapped as transcription units, ignoring occasional genes in the opposite orientation in a transcription unit unless there was high plausibility of expression (for example, rRNA genes or protein-coding genes with a clear localization). Transcription units comprising >20 protein-coding genes with localization data were analysed.

Predicted mitochondrial presequence and signal peptide targeting sequences were identified using TargetP-v2.0 (ref. ^88^).

### Orthology and selection pressures

Evidence for evolutionary trends and conservation in specific organisms was derived from predicted protein sequences from 102 genomes, with broad coverage of eukaryotes and comprehensive coverage of Discoba lineages. This was supplemented with ten transcriptomes from Excavata lineages with poor genomic coverage, from which protein sequences were predicted using TransDecoder v5.5.0 (LongOrfs)^89^ (Supplementary Table 4).

Orthologous groups were determined Orthofinder v2.3.12 (refs. ^90,91^) using default settings using diamond v2.0.5 and FastME 2.1.4. Reciprocal best BLAST (RBB) hits to *T*. *brucei* TREU927 proteins were identified using National Center for Biotechnology Information BLAST 2.9.0+ (ref. ^92^), reciprocal hits irrespective of forward and reverse search e-value and accepting reciprocal hits that identified a *T*. *brucei* gene with an identical sequence to the starting gene. For our analyses, a protein in a different species was defined as ‘the’ orthologue of a *T*. *brucei* gene if it was either the RBB or was the only orthogroup member in that species. We have used current bioinformatic approaches for all protein–protein orthologue analyses, but these are limited by the power of such computational comparative approaches.

Gain in complexity at different evolutionary distances was carried out using National Center for Biotechnology Information Taxonomy species classifications. We scored the proportion of proteins localizing to a particular organelle that had an orthologue in at least one species at that evolutionary distance (Supplementary Table 4) using a hypergeometric test to detect over-enrichment.

To determine the ratio of number of non-synonymous mutations (*K*_A_) to synonymous mutations (*K*_S_) RBB protein sequences were aligned using Clustal Omega^93^. The corresponding coding sequences were mapped to the protein sequence alignment and scored for identical codons (no mutation), synonymous mutation, non-symonymous mutation or indel mutation (alignment gap). *K*_A_/*K*_S_ was calculated per codon treating gaps as non-synonymous mutations. *K*_A_/*K*_S_ was calculated without any codon bias correction for *T*. *brucei* *brucei* TREU927 against each *Trypanozoon* (African trypanosome) species for each reciprocal best BLAST orthologue, and averaged.

### Human and yeast proteins

Human and yeast protein localizations were obtained from the respective project websites. For C-terminal whole-genome yeast tagging projects^2,94^, https://yeastgfp.yeastgenome.org/allOrfData.txt for *S*. *cerevisiae* and a custom web scraper from https://www2.riken.jp/SPD/01/01A01.html for *S.* *pombe* (both accessed December 2020). For the human antibody-based Human Protein Atlas/Cell Atlas project^4^, https://www.proteinatlas.org/download/subcellular_location.tsv (accessed September 2020). Annotation terms were remapped to the most similar *T*. *brucei* structure for comparison (Supplementary Table 5).

Evidence for involvement in human genetic disease was determined from OMIM^95,96^, accessed November 2018. Entries per gene were mapped to Ensembl gene identifications (IDs) (using the OMIM-provided mapping), and Ensembl gene IDs were mapped to Uniprot protein IDs (using the Uniprot protein mapping). Proteins were taken as involved in disease if the parent gene was annotated as associated or statistically linked with disease, involved with a known molecular mechanism or involved along with multiple genes.

### Deletion mutant generation and characterization

Clustered regularly interspaced short palindromic repeats (CRISPR)/Cas9 genome editing was used to delete candidate genes^97^. PCF TREU927 1339 cells that express T7 RNA polymerase, tet repressor and Cas9 were used^98^. Deletion primers and guide RNA primers were designed using the LeishGedit website and used to amplify constructs containing resistance markers with homology arms to the specific genes and specific guide RNAs (primer sequences in Supplementary Table 6). Plasmids pPOTv7-mNG-hyg and pPOTv7-mNG-bsr were used as the template for the deletion constructs. Gene deletion was confirmed by PCR from genomic DNA, using primers in the deleted gene ORF (Supplementary Table 6). Growth was monitored using a Z2 Coulter Counter. For growth curves, cell density was measured every 24 h over a 96 h period, with cells subcultured every 24 h to 2 × 10^6^ cells ml^−1^. Microscopy was carried out on live cells. Cells were washed two times with Dulbecco’s Modified Eagle Medium (phenol red free) and 500 ng ml^−1^ Hoechst 33342 was included in the first wash to stain the DNA^79^. Micrographs were captured on a Leica DM5500 B epifluorescence microscope with a 100 W mercury arc lamp and either a 63× NA1.4 oil immersion objective and an Andor Neo 5.5 sCMOS camera running in 16-bit high well capacity mode, using Micromanager^80^. mNG fluorescence was captured where applicable using the L5 filter cube, excitation 480/40 nm, dichroic 505 nm and emission 527/30 nm. Using these micrographs, the cell cycle staging for each cell line was analysed by categorizing cells on the basis of the number of kinetoplasts and nuclei.

### Electron microscopy

Cells were fixed briefly with 2.5% glutaraldehyde in medium, washed in phosphate-buffered aaline and then fixed for 1 h with 2.5% glutaraldehyde/4% formaldehyde in 0.1 M cacodylate buffer (pH 7.2). Samples were post-fixed in 1% osmium tetroxide/1.5% potassium ferricyanide in 0.1 M cacodylate buffer for 1 h, stained with 1% uranyl acetate/0.2% acetic acid overnight (at 4 °C) and then dehydrated in ethanol. Samples were infiltrated and embedded in TAAB 812 resin Hard (TAAB T030 kit). All sample preparation steps were performed at room temperature, unless stated otherwise. Ultrathin sections were post-stained with lead citrate for 5 min and imaged at 120 kV, in a JEM 1400 Flash transmission electron microscope (Jeol), equipped with a OneView camera (Gatan).

### Reporting summary

Further information on research design is available in the Nature Portfolio Reporting Summary linked to this article.

## Supplementary information

Reporting Summary

Supplementary Table Supplementary Tables 1–8.

## Figures and Tables

**Extended Data Fig. 2:**
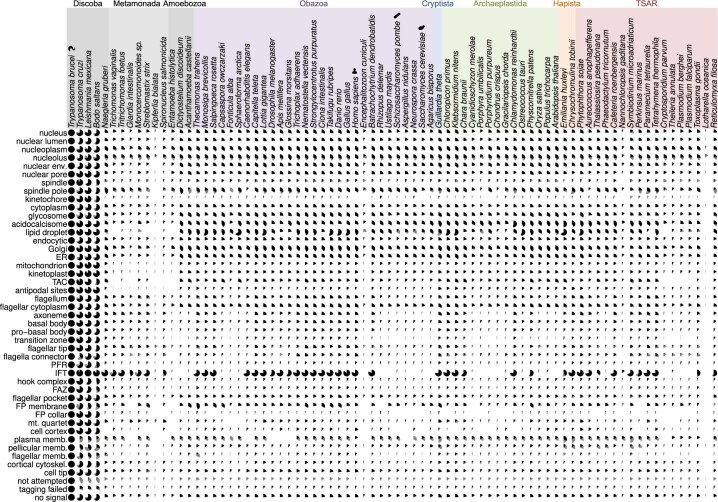
Extended mapping of the universally conserved features of eukaryotic organelles. Extended version of Fig. 2a. **a**. Presence of orthologs of *T. brucei* proteins, grouped by organelle, across eukaryotic life. Pies represent the proportion of proteins with a reciprocal best BLAST (RBB, black) or not an RBB but at least one orthogroup member (grey) in each species. Source data

## Data Availability

All microscopy data is available at Zenodo with one DOI per 96-well plate. All DOIs are listed by 96-well plate in Supplementary Table 7 and by gene ID in Supplementary Table 8. The master record is under 10.5281/zenodo.6862289 (ref. ^99^). Data can be browsed at http://tryptag.org and are incorporated in the *T*. *brucei* genome database at http://tritrypdb.org. Source data are provided with this paper.

## Source data

Source Data Fig. 1 Source data.
Source Data Fig. 2 Source data.
Source Data Fig. 3 Source data.
Source Data Fig. 4 Source data.
Source Data Fig. 5 Source data.
Source Data Extended Data Fig. 1 Source data.
Source Data Extended Data Fig. 2 Source data.
Source Data Extended Data Fig. 3 Source data.
Source Data Extended Data Fig. 4 Source data.
Source Data Extended Data Fig. 5 Source data.
Source Data Extended Data Fig. 6 Source data.
Source Data Extended Data Fig. 7 Source data.
Source Data Extended Data Fig. 8 Source data.
