# Supplementary material for: Genome-wide subcellular protein map for the flagellate parasite Trypanosoma brucei
Source: Nat Microbiol. 2023 Feb 20;8(3):533–47. doi: 10.1038/s41564-022-01295-6 (PMC9981465; doi:10.1038/s41564-022-01295-6)
Supplement: Supplementary file 1 — Reporting Summary [file 41564_2022_1295_MOESM1_ESM.pdf]

nature portfolio

Corresponding author(s): Richard Wheeler, Jack Sunter, Samuel Dean

Last updated by author(s): 27/10/2022

Reporting Summary

Nature Portfolio wishes to improve the reproducibility of the work that we publish. This form provides structure for consistency and transparency in reporting. For further information on Nature Portfolio policies, see our Editorial Policies and the Editorial Policy Checklist.

Statistics

For all statistical analyses, confirm that the following items are present in the figure legend, table legend, main text, or Methods section.

n/a	Confirmed	
☐	☒	The exact sample size (*n*) for each experimental group/condition, given as a discrete number and unit of measurement
☐	☒	A statement on whether measurements were taken from distinct samples or whether the same sample was measured repeatedly
☐	☒	The statistical test(s) used AND whether they are one- or two-sided<br>*Only common tests should be described solely by name; describe more complex techniques in the Methods section.*
☒	☐	A description of all covariates tested
☒	☐	A description of any assumptions or corrections, such as tests of normality and adjustment for multiple comparisons
☐	☒	A full description of the statistical parameters including central tendency (e.g. means) or other basic estimates (e.g. regression coefficient) AND variation (e.g. standard deviation) or associated estimates of uncertainty (e.g. confidence intervals)
☐	☒	For null hypothesis testing, the test statistic (e.g. *F*, *t*, *r*) with confidence intervals, effect sizes, degrees of freedom and *P* value noted<br>*Give P values as exact values whenever suitable.*
☒	☐	For Bayesian analysis, information on the choice of priors and Markov chain Monte Carlo settings
☒	☐	For hierarchical and complex designs, identification of the appropriate level for tests and full reporting of outcomes
☒	☐	Estimates of effect sizes (e.g. Cohen's *d*, Pearson's *r*), indicating how they were calculated

Our web collection on statistics for biologists contains articles on many of the points above.

Software and code

Policy information about availability of computer code

Data collection	Microscopy images were captured using Micromanager (no single version, updated over the course of data collection).
Data analysis	Microscopy images were analysed using ImageJ (1.52a). Custom image analysis was carried out using ImageJ scripts, provided in Zenodo DOI 10.5281/zenodo.6862289. Orthology was determined using Orthofinder (2.3.12), diamond (2.0.5) and FastME (2.1.4). Reciprocal best BLAST used NCBI BLAST (2.9.0). Kn/Ks analysis used Clustal Omega (1.2.4). Transcripts from transcriptomes were predicted using TransDecode (v5.5.0)

For manuscripts utilizing custom algorithms or software that are central to the research but not yet described in published literature, software must be made available to editors and reviewers. We strongly encourage code deposition in a community repository (e.g. GitHub). See the Nature Portfolio guidelines for submitting code & software for further information.

Data

Policy information about availability of data

All manuscripts must include a data availability statement. This statement should provide the following information, where applicable:

- Accession codes, unique identifiers, or web links for publicly available datasets
- A description of any restrictions on data availability
- For clinical datasets or third party data, please ensure that the statement adheres to our policy

All microscopy data is available at Zenodo with one DOI per 96 well plate. All DOIs are listed by 96 well plate in Table S7 and by gene ID in Table S8. The master

record is under DOI 10.5281/zenodo.6862289124. Data can be browsed at http://tryptag.org and is incorporated in the T. brucei genome database at http://tritrypdb.org.

Human research participants

Policy information about studies involving human research participants and Sex and Gender in Research.

Reporting on sex and gender	N/A
Population characteristics	N/A
Recruitment	N/A
Ethics oversight	N/A

Note that full information on the approval of the study protocol must also be provided in the manuscript.

Field-specific reporting

Please select the one below that is the best fit for your research. If you are not sure, read the appropriate sections before making your selection.

☒ Life sciences ☐ Behavioural & social sciences ☐ Ecological, evolutionary & environmental sciences

For a reference copy of the document with all sections, see nature.com/documents/nr-reporting-summary-flat.pdf

Life sciences study design

All studies must disclose on these points even when the disclosure is negative.

Sample size	Sample sizes for the protein localisation resource were not predetermined, analysis was based on ~>250 cells per cell line - the maximum that could be visualised for genome-wide resource generation within microscopy time available. This effectively samples the estimated 5-20 clones giving rise to each non-clonal population and captures rare (~5%) cell cycle stages, as described in the text. Sample sizes for mutant analysis were established according to best practices within the field and are similar to recently published work, three independent clonal mutant cell lines.
Data exclusions	No data were excluded.
Replication	For the protein localisation resource, genome-wide protein tagging was carried out once. Replication involved tagging at the N and C terminus wherever possible, as described in the text. Deletion mutant phenotype was confirmed by generation of 4 independent cell lines.
Randomization	Randomisation is not relevant as we determined the subcellular localisation of all qualifying genes in the genome. It is not possible to randomly subsample when analysing all genes in the genome.
Blinding	Protein localisation annotation was carried out blinded to the gene IDs and names. Analysis of light micrographs of mutant cell lines were blinded to the identity of the cell line.

Reporting for specific materials, systems and methods

We require information from authors about some types of materials, experimental systems and methods used in many studies. Here, indicate whether each material, system or method listed is relevant to your study. If you are not sure if a list item applies to your research, read the appropriate section before selecting a response.

Materials & experimental systems

n/a	Involved in the study	
☒	☐	Antibodies
☐	☒	Eukaryotic cell lines
☒	☐	Palaeontology and archaeology
☒	☐	Animals and other organisms
☒	☐	Clinical data
☒	☐	Dual use research of concern

Methods

n/a	Involved in the study	
☒	☐	ChIP-seq
☒	☐	Flow cytometry
☒	☐	MRI-based neuroimaging

Eukaryotic cell lines

Policy information about cell lines and Sex and Gender in Research

Cell line source(s)	Long-term laboratory stocks of Trypanosoma brucei cell lines.
Authentication	Genome and mRNA sequencing prior to start of the protein localisation resource generation.
Mycoplasma contamination	Cell lines were monitored for contamination, including mycoplasma contamination, through DNA staining and microscopy during data capture.
Commonly misidentified lines (See ICLAC register)	N/A
